# Towards a European End-of-Life Regulation: A Necessary Analysis

**DOI:** 10.3390/healthcare13020130

**Published:** 2025-01-11

**Authors:** Francesco Orsini, Andrea Cioffi, Luigi Cipolloni, Maria Antonella Bosco, Chiara Fabrello, Camilla Cecannecchia, Stefania De Simone

**Affiliations:** Department of Clinical and Experimental Medicine, Section of Forensic Medicine, University of Foggia, 71122 Foggia, Italy; francesco.orsini@unifg.it (F.O.); andrea.cioffi@unifg.it (A.C.); maria.bosco@unifg.it (M.A.B.); chiara.fabrello@unifg.it (C.F.); camilla.cecannecchia@unifg.it (C.C.); stefania.desimone@unifg.it (S.D.S.)

**Keywords:** end of life, euthanasia, advance directives, informed consent, assisted suicide, bioethics

## Abstract

**Background/Objectives:** Biomedical progress has extended the lifespan of patients with incurable diseases, sparking debates about their desire to live under certain conditions. This study examines the ethical and legal challenges surrounding end-of-life issues in Europe, including informed consent, the refusal of treatment, the right to health, self-determination, advance directives, assisted suicide, and euthanasia. European countries exhibit different interpretations and regulations of these practices, leading to patient “migrations” seeking favorable legal environments. **Methods:** This study analyzes end-of-life legislation across European countries in a comparative and qualitative way, highlighting differences, commonalities, and the potential for uniform regulation. The data were collected from the literature published between 2000 and 2024, focusing on the EU member states, Switzerland, and the United Kingdom. **Results:** The examination of the norms governing end-of-life practices in various European countries revealed significant differences in legislative frameworks, reflecting diverse cultural, ethical, and legal perspectives. These variations have led to patient migrations in search of suitable legal environments to end their lives with dignity. **Conclusions:** This study highlights the need for a harmonized approach to end-of-life legislation in Europe to ensure equitable access to end-of-life care and to uphold human dignity. Continuous legal updates and comparative studies are essential to balance medical advancements with ethical considerations. The findings emphasize the importance of autonomy and self-determination, which are fundamental human rights that should be respected in the context of end-of-life decisions.

## 1. Introduction

Biomedical progress has enabled the prolongation of life for patients suffering from incurable diseases. These innovations have sparked debates about the patients’ actual willingness to remain alive under certain conditions [1]. Consequently, various European states have questioned the limits of protecting the right to life, particularly in severely ill patients. The end-of-life issue encompasses several themes: informed consent, the refusal of treatment, the right to health, self-determination, advance directives, assisted suicide, and euthanasia [2]. Assisted suicide refers to the volition to terminate one’s life through the self-administration of lethal drugs. Usually, a physician prescribes or supplies the drug, which the patient independently administers to induce death (medically assisted suicide) [3]. Euthanasia is the deliberate act of ending the life of a person suffering from an irreversible illness and intolerable pain, carried out at their explicit request [4]. Furthermore, there is the explicit condition that the patient must be suffering from unbearable physical, emotional, or spiritual pain and that the affliction is uncontrollable with conventional measures such as medical treatments, analgesics, and other palliative care: the objective of euthanasia is to alleviate this suffering. This is different from deep sedation, which is included in palliative medicine and uses the intentional administration of drugs to reduce or nullify the patient’s consciousness to alleviate intolerable physical or mental symptoms that are refractory to any treatment [5,6,7]; therefore, deep sedation does not fall within the scope of a euthanasia procedure, because, having the purpose of eliminating pain and suffering, it is not an action or an omission that, even in its intentions, aims to cause death.

Additionally, euthanasia can be categorized into two types: active and passive. Active euthanasia involves a direct action that leads to the patient’s death, such as the administration of lethal drugs. Passive euthanasia, on the other hand, involves the withdrawal or withholding of life-sustaining treatments, allowing the illness to take its natural course [8].

Regarding euthanasia for minors, the Netherlands and Belgium are the only European countries that permit it. Belgium has allowed euthanasia for terminally ill minors without any age limit since 2014, while the Netherlands permits it for terminally ill children aged 12 and older [9].

Advance directives, also known as living wills or advance decisions, are legal documents that allow a person of legal age and with the capacity to make decisions to express their healthcare preferences in advance, in case they become unable to make decisions for themselves in the future. These directives include decisions regarding diagnostic procedures, therapeutic choices, and individual healthcare treatments [10].

Informed consent in Europe is a fundamental principle that requires patients to be adequately informed about proposed medical treatments and to give their free and informed consent before such treatments can be carried out. This principle ensures respect for the autonomy and rights of patients, allowing them to actively participate in decisions regarding their health [11].

For informed consent to be valid, it must be based on clear and comprehensible information about the diagnosis, the nature of the treatment, the risks and benefits, the available alternatives, and the consequences of refusing the treatment. The patients must demonstrate that they understand this information and give their consent voluntarily, without coercion or deception. Additionally, they must have the mental capacity to make informed decisions. Informed consent is usually documented through the signing of a form that confirms the patient has been informed and has agreed to the treatment. Although specific laws and regulations may vary between European countries, these general principles are shared across the continent [12,13].

In European countries, the interpretation of such definitions is not always consistent across legislative and judicial authorities. Over the years, provisions with ambivalent meanings have followed one another [14]. Some of them have an international and supranational impact; others have a merely declarative character: they do not have a legally binding effect despite becoming a challenging point of reference in the development of further international conventions in the European context, as well as national laws on the matter within the member countries [15]. Recommendation 1418 [16] addresses the issue of the protection of dignity for terminally ill patients. It states that, to guarantee the dignity of the sick, it will be necessary to make treatment available to them in palliative care that can be accessed under equitable conditions. This case aims to offer specifically trained professionals to both family members and patients in this field, guaranteeing true, complete, and proportionate information that allows the patient to decide on the treatment and type of care that is best for them, respecting the right to self-determination [17]. The Recommendation reiterates that in no case should death be proposed as one of the possibilities for alternative treatments. Member states are asked not to consider the wish of a terminally ill patient to die as a legally recognized right to die through the intervention of another person, nor a justification for carrying out practices aimed at causing death. To date, the European Parliament has not passed a law to regulate the end-of-life issue, but almost all the individual states have approved laws that often provide diametrically opposed rules on some aspects of the problem. The differences in laws and procedures observed across various European countries have generated the phenomena of “migrations” of patients attempting to end their existence by seeking the regulatory situation most suitable to their condition [18]. Therefore, it is valuable to examine the norms governing end-of-life practices in various European countries. This examination aims to understand their differences, highlight common points, and evaluate the possibility of achieving uniform regulation on this issue.

## 2. Materials and Methods

This study adopts a comparative and qualitative approach to analyze the different end-of-life legislations in European countries. The aim is to identify the main differences and similarities in the regulations and to evaluate the impact of such laws on medical practice and patients’ rights.

A review of currently published studies was performed following the PRISMA criteria [19]. The literature search was conducted via PubMed and Scopus, and it was carried out for articles published from 2000 onward using the following keyword terms: ((assisted suicide) AND (euthanasia) AND (end of life)). Additionally, data were collected through a systematic review of online legislative documents, government reports, and publications from non-governmental organizations. Only the documents published between 2000 and 2024 were included to ensure the relevance and currency of the information. This study included the member countries of the European Union, Switzerland, and the United Kingdom. The countries with incomplete or unavailable legislation were excluded. Additionally, the articles that did not specifically address the topic of the end of life were excluded.

**Figure 1:**
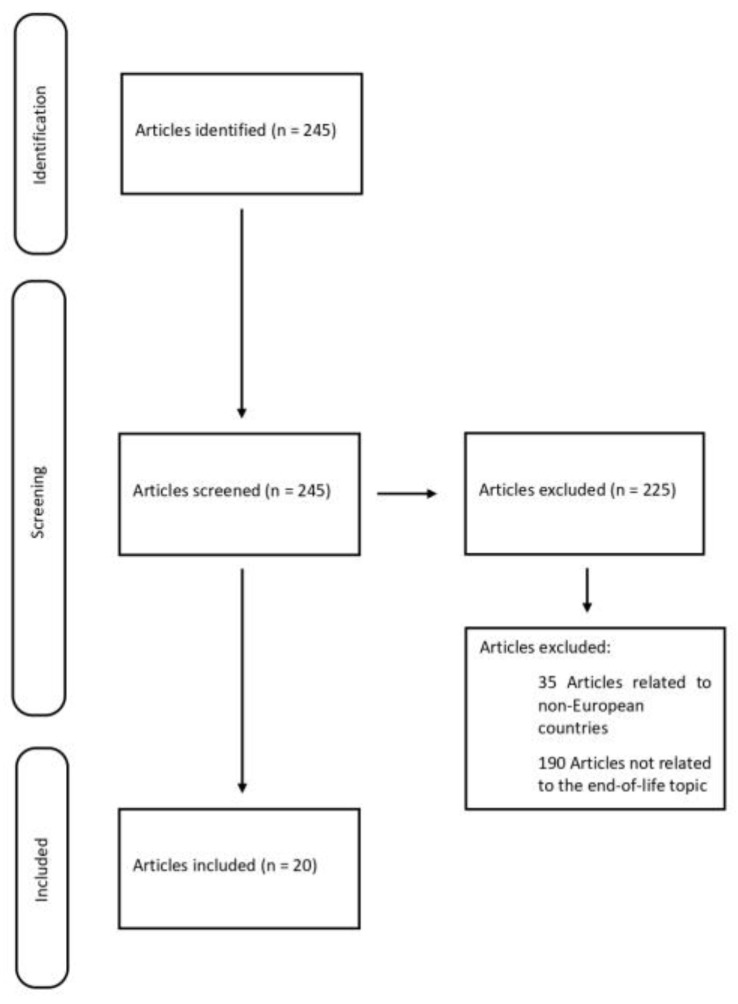
Search strategy following PRISMA criteria.

The initial electronic data search yielded a total of 245 potentially relevant studies on PubMed and Scopus. The articles were carefully evaluated based on their titles, abstracts, and texts. The implementation of these procedures resulted in only 20 eligible papers for inclusion from the initial 245 articles within the search (Figure 1).

## 3. Results

Several European legal systems were analyzed (Table 1). It should be noted that Bulgaria, Cyprus, Croatia, Greece, and Estonia prohibit any form of euthanasia and assisted suicide.

### 3.1. Netherlands

The Dutch law of 10 April 2001 regulates living wills, defining assisted suicide as the act of intentionally helping another person to commit suicide or providing them with the means to conduct it [20]. The Dutch legal system excludes the liability of a physician who causes the death of a consenting patient or assists in suicide, provided that the criteria of due care are met. According to these criteria, the request for euthanasia must come from the patient themselves and must be a voluntary and well-considered decision. This means that the patient must request euthanasia without being influenced by others and must have reflected on this choice for a long time. The request must be made clearly and consciously, without any external pressure. The patient must also suffer unbearably and without any hope of improvement [21]. Suffering can be both physical and psychological, but it must be such as to make life intolerable. The physician must carefully assess the patient’s situation to ensure that the suffering is indeed unbearable and that there are no possibilities for improvement. It is essential that the patient be fully informed about their condition. The physician must explain in detail to the patient their medical situation, prospects, and all available treatment options, including palliative care. Another important criterion is consultation with a second, independent physician. The physician intending to perform euthanasia must consult an independent colleague who will examine the patient and confirm that the legal criteria for euthanasia are met. This consultation is necessary to ensure that the decision is well-founded and not made hastily [22]. The physician must also be convinced that there are no reasonable alternatives to alleviate the patient’s suffering. If there are other treatment options that could improve the patient’s quality of life, these must be considered before proceeding with euthanasia. When performing euthanasia, the procedure must be carried out with the utmost medical care. This means that safe and accepted methods must be used to ensure that death occurs painlessly and with dignity. After performing euthanasia, the physician must document each stage of the process in detail. This documentation includes the reasons for the patient’s request, the consultations carried out, and the methods used. Finally, the case must be reported to a regional euthanasia review committee, which will assess whether all the legal criteria have been met. These criteria have been developed to ensure that euthanasia is practiced only under strictly controlled circumstances, thereby ensuring that the rights and dignity of the patients are respected and that the physicians act in accordance with ethical and legal standards. Article 2 of the Dutch law allows the application of accuracy criteria, even if the patient cannot express their will, if they have issued a written advance directive after the age of 16. For the age group of 16–18 years, the request can be executed if the parents or guardian are involved. Individuals aged between 12 and 15 years require parental permission [23].

### 3.2. Belgium

The Belgian law of 28 May 2002 legalized euthanasia. Unlike some other countries, Belgium makes no legal distinction between euthanasia and physician-assisted suicide, though in practice euthanasia is far more common [24]. The fundamental principle of this law is the individual’s self-determination. According to this law, euthanasia is the act performed by a third party who deliberately ends a person’s life at their request [25]. The physician who performs euthanasia does not commit any violation if they comply with the conditions and procedures prescribed by the law. The patient must be of legal age or an emancipated minor and mentally competent at the time of the request. The request must be made voluntarily, thoughtfully, and repeatedly and not be induced by external pressures [20]. The patient must be in a hopeless medical condition with unbearable and intractable physical or psychological suffering, resulting from a severe and incurable chronic disease [26]. Additionally, the physician must inform the patient about their health condition and life expectancy, discuss the patient’s request for euthanasia, and present all the remaining therapeutic options, as well as the opportunities offered by palliative care and their effects [27]. To proceed with euthanasia, except in cases of urgency, the opinion of two physicians is required, and at least one month must elapse between the patient’s written request and the euthanasia procedure. In 2014, the law was extended to include terminally ill minors [28], although a 2015 ruling by the Constitutional Court required that, for minors, an independent psychiatrist or child psychologist, separate from both the attending physician and the patient and their legal representatives, be involved: this was to protect the most vulnerable individuals in order to prevent abuse and ensure their right to life and physical integrity [29].

### 3.3. Denmark

Denmark has developed a complex and nuanced approach to end-of-life issues, seeking to balance respect for patient autonomy with the protection of human life. This balance is reflected in a series of laws and regulations. In 1998, the country took a significant step with the enactment of the law on the “living will” (Lov om patienters retsstilling). This legislation allowed citizens to express their wishes regarding medical treatments in case they can no longer exercise their right to self-determination. To facilitate this process, an electronic database called “Patients’ Register” or “eHealth” was established, which stores citizens’ advance directives [30]. Thanks to this law, Danes who have filed a living will can request the cessation of medical care and life-sustaining treatments in case of incurable illness or serious accident, avoiding being kept alive artificially. Furthermore, the right of patients to refuse treatments that only serve to prolong life without offering any prospect of cure, improvement, or relief has been recognized. It is important to note that the application of these wishes varies depending on the circumstances. In the case of terminal illness, the patient’s wishes are binding on healthcare personnel. However, in other situations, such as old age, accident, or cardiac arrest, these wishes are considered indicative and must be evaluated in the context of the specific situation [31]. The medical profession in Denmark is guided by the code of ethics “Lægeforeningens Etiske Regler”, which establishes the ethical principles for physicians. This code, along with the position of the Danish Medical Association, maintains a clear prohibition of active euthanasia, emphasizing that physicians must act in the patient’s interest and respect human life. However, in line with existing legislation, the code recognizes the right of patients to refuse life-prolonging treatments and allows palliative sedation in cases of extreme suffering [32].

### 3.4. Germany

The introduction of “patient directives” (Patientenverfügungen) in the German legal system dates back to the late 1970s. However, it was only in 2003 that the Federal Court of Justice (BGH) established through a ruling the legitimacy and binding nature of advance directives [33]. The BGH affirmed that the dignity of the person requires respect for the right to self-determination, even when the patient is no longer able to decide. In the absence of a specific declaration, consent to or refusal of treatments must be reconstructed based on the presumed will of the patient, considering their worldview and values. The ruling of 17 March 2003, therefore, confirmed the legitimacy and binding nature of advance directives. Only in 2009 did the “Third Law for the Amendment of the Fiduciary Relationship Regulation” recognize the living will at a legislative level and not just at the jurisprudential one. The judgment of 17 March 2003 (BGHZ 154, 205) establishes that, if a patient is unable to give consent and their illness is irreversible, life-sustaining measures must be avoided if they are contrary to the previously expressed will of the patient [34]. This is because human dignity requires respecting the right to self-determination, even when the patient is no longer able to make conscious decisions. If it is not possible to ascertain the patient’s will, the admissibility of measures can be assessed according to the presumed will of the patient, based on their previously expressed values and beliefs. The current law allows a competent person to declare in writing their consent to or refusal of medical treatments in case of future incapacity [35]. The fiduciary must assess whether these directives correspond to the patient’s current medical situation. If so, the consent is valid. If not, the fiduciary must express consent to or reconstruct the patient’s will based on concrete indications. These rules apply regardless of the patient’s illness. The physician discusses the measures with the fiduciary, considering the patient’s will and consulting third parties connected to the patient. The fiduciary’s consent/refusal requires the judge’s authorization in case of the imminent danger of death, unless the physician and fiduciary agree that it corresponds to the patient’s will. German legislation balances the values of personal freedom and dignity, the precautionary principle, and the importance of medical professionalism. The German Bundestag approved the bill no. 18/5373 on 6 November 2015: pursuant to the new article, organized assistance to suicide as a permanent medical or commercial offer is punishable by a fine or imprisonment for up to three years. Therefore, commercial (geschäftsmäßig) or association-based assistance to suicide is prohibited, while case-by-case decisions are not explicitly covered by the criminal prohibition. However, the law was unclear on whether a consulted physician would be considered a commercial partner due to the payment for the consultation. This ambiguity led to a discussion between Parliament and the court. The German Federal Constitutional Court ruled in February 2020 that the prohibition of assisted suicide services was unconstitutional, as it infringed upon the fundamental rights and principles enshrined in the German constitution. The court emphasized that, while Parliament could pass laws on preventing suicide and increasing palliative care, it was not entitled to affect the impunity of assisted suicide. The court’s decision highlighted the need for clearer legislation on the matter [36].

### 3.5. Spain

Spain officially legalized euthanasia on 25 June 2021, with the enactment of the Organic Law for the Regulation of Euthanasia [37]. The law is divided into six chapters. Chapter IV establishes respect for the patient’s autonomy. Article 11, paragraph 1, provides that an adult, capable, and free person may express in advance, through a living will (advance directives), their will in case they find themselves in conditions where they are unable to express their wishes regarding the treatments or therapies they intend to undergo or the fate of their body and organs. The individual can also appoint a representative who, in the event of subsequent incapacity, would act as an interlocutor with the physician or the healthcare team to execute such advance directives. Paragraph 3, however, prohibits the implementation of advance directives contrary to the legal system and the lege artis and renders the instructions ineffective if situations not corresponding to those foreseen by the interested party at the time of their formulation occur. Finally, paragraph 4 provided that such advance declarations can be revoked at any time. All advance directives are collected in a National Register [38]. On 18 March 2021, the Spanish House of Representatives approved law no. 3/2021 (the Organic Law on the Regulation of Euthanasia), which regulates euthanasia, defining it as a true right to request and obtain the necessary help to die [39]. The law recognizes, based on an autonomous decision and informed consent, the right to request and receive assistance to end one’s life. To make the request, the law requires that the individual must be of Spanish nationality and of legal age; be capable of understanding and willing and conscious at the time of the decision or, alternatively, have issued advance directives; be informed in writing about their clinical situation and possible alternatives, including palliative care; have made two written requests for assistance to die, at least 15 days apart (except in cases of particular necessity and urgency); be suffering from a serious and incurable disease or a serious, chronic, and disabling disease, as defined by the law and certified by competent physicians; and have signed the informed consent to receive the assistance to die. The law contains a detailed description of the procedures to be followed by the physician and provides a double system of controls by the competent control and evaluation commission. This assistance can be carried out in two ways: (1) the direct administration of a substance to the patient by the competent healthcare professional; (2) the prescription or provision by the healthcare professional of a substance to the patient, so that the patient can self-administer it to cause their own death. There is subsequent control over the assistance procedure: the physician must deliver two documents to the commission within 5 days of the patient’s death. The first contains all the personal data relating to the patient and the physician, while the second relates to the clinical history and the procedure followed. The commission initially analyzes only the second document to maintain anonymity and proceeds to verify the first only in case of doubt: if irregularities are found, it may order the healthcare center to open an investigation (Articles 12 and 18). The law specifies that assistance to die will be fully covered by the national health system and access to the procedure must be guaranteed to all, respecting privacy and confidentiality; it also allows physicians to exercise the right to conscientious objection, specifying that this must never prejudice access to and quality of care. The traditional distinction between euthanasia and assisted suicide is thus eliminated, emphasizing the central core: dying on request. Most notably, the Spanish law uses a term typical of treatments carried out by a healthcare professional, “Service”, where the treatment is “assistance to die”.

### 3.6. Portugal

The Portuguese law regarding palliative care, known as the “Lei de Bases dos Cuidados Paliativos”, was approved in 2012 and established the right of access to palliative care for all Portuguese citizens [40]. The law defines the principles and objectives of palliative care, establishes the rights and duties of patients, and sets up a national palliative care system. This law states that palliative care is a right for all Portuguese citizens; establishes that palliative care must be universally accessible, free of charge, and of high quality; and defines the objectives of palliative care, including pain and suffering relief, quality of life improvement, and support for patients and their families. Furthermore, this law enshrines the right of patients to refuse palliative care, guarantees the right of patients to be fully informed about their health condition and treatment options, and establishes the duty of healthcare professionals to respect the patient’s wishes and preferences. The law has also established a national network of services and facilities dedicated to palliative care.

Regarding advance directives, the advance directive law in Portugal, enacted in 2012 (law no. 25/2012 of 16 July), allows individuals to create living wills or appoint a healthcare attorney. This ensures that their medical treatment preferences are respected even if they become unable to communicate them. The law emphasizes autonomy and informed consent, providing a means for individuals to express their wishes regarding medical care in advance [41].

In 2021, the Portuguese Parliament approved a law on euthanasia and medically assisted suicide, which came into force in 2022 [42]. This law allows adult patients, residing in Portugal, suffering from a serious, incurable, and irreversible disease that causes them great suffering, to request euthanasia or medically assisted suicide under specific conditions. Minors and patients with mental disorders or dementia are explicitly excluded. To request euthanasia or medically assisted suicide, the patient must submit a written and signed request, confirmed after a two-week reflection period. At least two physicians, including one specialist in the patient’s pathology, must be involved to verify the requirements. A psychological evaluation is also required to ascertain the patient’s decision-making capacity. All the steps of the procedure must be documented in detail. Physicians are obliged to thoroughly inform the patient about their condition, therapeutic options, and the consequences of euthanasia/assisted suicide. The patient must give their free and informed consent at every stage of the procedure. Moreover, criminal penalties are provided for healthcare professionals who do not comply with the law.

### 3.7. Austria

The Austrian federal law no. 55/2006 regulates the “Patientenverfügung” (patient directive). The physician must respect the patient’s decision, even if the refused treatment is considered life-saving by medical science and even if the physician disagrees with the patient’s choice, as the duty of care does not prevail over the patient’s autonomy. Active assistance to the patient to die or contributing to causing their death was prohibited. Following the intervention of the Constitutional Court in 2020 and the legislator in 2021, assistance in suicide is allowed under certain conditions. The Austrian Federal Constitutional Court declared the provision that criminally punishes assistance in suicide unconstitutional, insofar as it provides that assistance to suicide by a third party may constitute a crime, because it contradicts the principle of self-determination. However, consensual homicide remains prohibited. Law no. 242/2021 of 31 December 2021 [43] defines its scope and purposes: it is applicable only to Austrian citizens or those who have their habitual residence in Austria and aims to regulate the requirements and effectiveness of advance directives resulting from a permanent, free, and self-determined decision (art. 1, sec. 1. 1, §1). The law does not impose any obligation to aid suicide (including the administration of drugs, medical counseling, or the drafting of the declaration of the intention to access such a procedure). However, those who provide such assistance (pharmacists, physicians, or lawyers) cannot be prosecuted for these actions (art. 1 sec. 1, §2). It is a declaration of will by which a person communicates their permanent, free, and self-determined decision to end their life, while assistance is defined as physical support in implementing measures that end life. The Austrian law provides for the role of a guarantor (Dokumentierende Person—literally, a “documenting person”) and defines the “preparation” as a lethal dose of sodium pentobarbital or another agent specified by prescription that ends the person’s life. It can be implemented in the case of terminal or severe and permanent illness with persistent symptoms, whose consequences permanently impair the entire lifestyle of the affected person, causing a state of suffering that cannot be otherwise avoided by the person concerned. It must be preceded by the declarations of two physicians, including a palliative care specialist, confirming that the person who wants to die has expressed a free and self-determined decision and indicating the actual existence of the requirements required by law. The implementation of the expressed will cannot take place before twelve weeks from the drafting of the declaration by the aforementioned two physicians, which is reduced to two weeks in the case of terminal illness, and becomes ineffective in case of revocation or if more than a year has passed since its drafting. The declaration of will is stored in an electronic register kept by the Federal Minister of Health. The law has provided for changes to the law on narcotic substances to ensure the application of the assisted suicide procedure as amended.

### 3.8. Switzerland

The Swiss Criminal Code (Articles 111, 113, and 114) forbids the intentional administration by a health professional or another person of a substance that causes death to a patient, even if aimed at reducing the patient’s suffering. The use of substances (e.g., morphine) to alleviate suffering, which may—as a side effect—shorten life, is accepted. This kind of euthanasia is not explicitly regulated by the current Criminal Code. However, it is permitted in principle according to the Euthanasia Guidelines of the Swiss Academy of Medical Sciences [44], which also consider this type of euthanasia to be permissible. Finally, Article 115 of the Swiss Criminal Code punishes those who assist or incite suicide for economic reasons, while those who do so for reasons of compassion, altruism, and solidarity are not punishable (Article 115 of the Swiss Criminal Code) [45]. Active euthanasia, however, is not allowed under any circumstances. Article 370 of the Swiss Civil Code allows anyone who is capable of discernment to decide on the medical treatments to be undergone in case they become incapable of discernment [46]. Every individual has the right to refuse medical treatment after a thorough examination by specialists, regardless of their health condition [47]. For those who are no longer able to express their opinion due to illness or accident, Swiss law provides for a “living will”. This document allows someone to establish in writing their desire for medical treatment in the event that they lose the ability to decide and communicate. It is possible to appoint one or more persons to be involved in the decision-making process on behalf of the individual. The directives must be written, dated, and signed and may be recorded on the health card with mention of the place where they are deposited.

### 3.9. United Kingdom

The United Kingdom has developed over time a legislative framework that seeks to balance respect for patient autonomy, the protection of vulnerable individuals, and ethical considerations pertaining to medical practice. In the United Kingdom, patients with mental capacity possess the legal right to refuse any medical treatment, including life-sustaining interventions. This principle has been codified in the Mental Capacity Act 2005 [48]. The Mental Capacity Act 2005 introduced the concept of “advance decisions”, which enable individuals to specify in advance which medical treatments they wish to refuse if they lose decision-making capacity [49]. The United Kingdom has developed a complex regulatory framework concerning end-of-life issues. The legislation allows the appointment of a proxy decision-maker who can make healthcare decisions on behalf of an individual who has lost their decision-making capacity [50]. Active euthanasia and assisted suicide are illegal in the United Kingdom, pursuant to the Suicide Act 1961 [51]. However, there have been numerous attempts to amend this legislation, including the Assisted Dying Bill, which was debated in Parliament but not enacted [52]. Medical practice in the United Kingdom recognizes the “doctrine of double effect”, which permits the administration of analgesics that may hasten death, provided that the primary intention is to alleviate pain rather than cause death. In certain circumstances, medical practitioners may lawfully withdraw or withhold life-sustaining treatments if deemed not to be in the patient’s best interests. These decisions are governed by professional guidelines and may be subject to judicial review in cases of dispute [53]. Significant case law includes Airedale NHS Trust v Bland [1993], which established that life-sustaining treatments may be lawfully withdrawn for patients in a persistent vegetative state [54]. Another notable case is R (on the application of Nicklinson) v Ministry of Justice [2014], which upheld the prohibition on assisted suicide but urged Parliament to reconsider the issue (R (on the application of Nicklinson and another) v Ministry of Justice [2014] KSC 38). In conclusion, end-of-life legislation in the United Kingdom is predicated upon a delicate balance between respect for patient autonomy and the protection of vulnerable individuals. While practices such as treatment refusal and advance decisions are well-established, euthanasia and assisted suicide remain the subject of ongoing ethical and legal debate.

### 3.10. France

The French law no. 2016-87 of 2 February 2016, known as the “Claeys–Léonetti Law”, guarantees patients the right to refuse any treatment, including life-sustaining measures, without permitting actions intended to cause or hasten death [55]. For patients unable to express consent or dissent, the French law utilizes advance directives (directives anticipées) [56]. Advance directives in France require assessing their consistency with the patient’s current situation. According to Article L1111-11, advance directives can be disregarded if they are “manifestly inappropriate” or “not in accordance with the medical situation”. The attending physician, with the agreement of the trusted person designated by the patient, may choose to ignore these directives. Additionally, if new therapies emerge after the directives are signed, they may also be disregarded.

The trusted person helps healthcare professionals interpret the patient’s will, especially when communication is impossible. Physicians must reconstruct the patient’s wishes, often expressed before illness. French law mandates a collegial procedure for deciding on the suspension of care, regulated by the French Code of Medical Ethics. Article R. 4127-37-2 requires this decision to follow the collegial procedure and consider advance directives or, in their absence, consult the trusted person or family. This process begins with a consultation between the attending physician and medical team, requiring a reasoned opinion from an independent physician. The trusted person or family members do not participate in the procedure but provide testimony. Ultimately, the physician decides on treatment suspension after consulting those best suited to represent the patient’s will.

In France, physicians suspending treatments must align decisions with the patient’s presumed will, without a specific degree of evidentiary certainty. The law highlights the medical aspect within the collegial procedure, regulated by normative sources. The Code of Medical Ethics grants physicians significant authority in suspending life-sustaining treatments. The 2016 Claeys–Léonetti law enables patients to express advance directives and refuse therapeutic obstinacy, strengthens access to palliative care, and permits deep sedation until death for terminal patients. The French legal system does not specify “assistance to suicide” but addresses the propaganda or advertising of suicide methods. Cases of assistance to suicide by non-health professionals are rare in French jurisprudence, which includes numerous end-of-life issues.

The Kouchner law of 1999, which focuses on free and informed consent, has led to significant developments in French law, recognizing the value of life as more accessible to the individual and showing a form of tolerance towards passive euthanasia. Subsequently, the Claeys–Léonetti law legalized, by the decision of the medical team, the discontinuation of “useless and disproportionate” treatments, even in cases where the patient’s will cannot be known. The French Conseil d’État has emphasized that end-of-life regulation is subject to four guiding principles. The first guiding principle is equitable access to the necessary care for one’s health condition, based on a fundamental right corresponding to the protection of health enshrined in the preamble of the 1946 Constitution of the Fourth French Republic. The patient must have a certain equality of access to palliative care, and this principle is crystallized in the law of 9 June 1999. Equality of access to palliative care means that all individuals, regardless of their socioeconomic, geographic, or personal condition, must have the opportunity to receive adequate palliative care to alleviate suffering in cases of serious or terminal illness [56]. The second guiding principle prohibits, as indicated in Article L 110-5-1 of the French Public Health Code, therapeutic obstinacy. It is not possible to administer care or treatments that constitute obstinacy. The Claeys–Léonetti law allows the discontinuation of artificial hydration and nutrition if they are useless, disproportionate, or have no other objective than the artificial maintenance of life. The legislator indicates that, when the patient is unable to express their will, the physician must still try to interpret or seek it according to the provisions of Article 1111-12 of the French Public Health Code. Thus, the decision to discontinue care must be adopted only within the framework of a collegial procedure that provides for an advisory opinion from another physician [57]. It is important to clarify that discontinuing treatments does not mean stopping care. This often aims to protect the dignity of the dying person through palliative care. The Claeys–Léonetti law permits deep sedation until death, combined with analgesia and the cessation of life-sustaining treatments. Article L. 1110-5-3 allows pain-relief treatments even if they may accelerate death. Patients can accept, refuse, or discontinue treatment, even if it leads to death. The French legislator introduced advance directives in 2005 to enhance patient autonomy, allowing them to make advance decisions about treatments and appoint a trustee. Decree no. 2006-119 requires physicians to respect these directives, prioritizing the trustee’s opinion. Article R.4127-38 of the French Public Health Code prohibits active euthanasia. The patient’s right to refuse treatments or accept palliative care does not legalize assisted suicide or active euthanasia. French law distinguishes between ceasing treatments (leading to natural death) and actively causing death, which remains prohibited. Therefore, interpreting this as the indirect legalization of euthanasia is incorrect as the current legislation deems such acts criminal offenses.

In this regard, the French Constitutional Court has declared constitutional the provision that gives physicians the possibility to refuse to perform acts of therapeutic obstinacy even in the absence of the expressed will of the patient who is unable to express it after the completion of the collegial evaluation procedure [58]. Similarly, the French Constitutional Court has declared the provision constitutional [57] that grants the physician the authority not to apply advance directives that are manifestly inappropriate or not in accordance with the patient’s medical situation [59]. The French legal system categorically differentiates between allowing death to occur naturally after the cessation of treatment and actions that directly cause death. In the absence of the true regulation of assisted suicide and active euthanasia, all behaviors aimed at helping a person commit an act of suicide are subject to criminal prosecution. Finally, it is noted that the absence of a crime of “assistance to suicide” limits such situations, and, should behaviors fall into this category, the French criminal justice system tends to be highly understanding.

### 3.11. Italy

The Italian law no. 219 of 22 December 2017 guarantees patients the right to refuse any treatment, including life-sustaining measures, without permitting actions intended to cause or accelerate death [55]. For patients unable to express consent or dissent, Italian law utilizes an advance healthcare directive (known as a DAT in Italian) [60]. This tool serves to reconstruct the previously expressed wishes of the patient, but it also requires an evaluation of the consistency of those wishes with the patient’s current situation. Italian law provides exceptions to the binding nature of advance healthcare directives. Indeed, Article 4, paragraph 5, of Italian law no. 219/2017 excludes the applicability of advance healthcare directives that are “incongruous”. Therefore, the attending physician, in agreement with the trusted person designated by the patient, may disregard them [61]. Advance healthcare directives may also be disregarded if new therapies have emerged after their signing. The role of the trusted person is crucial in assisting healthcare professionals to correctly interpret the patient’s will, especially when the patient cannot express themselves. The physician must reconstruct the patient’s will, often expressed before the onset of the illness when the current situation was unforeseeable. This process aims to avoid the risk of not respecting the vulnerable patient’s will or imposing someone else’s will on them [62]. Italian law designates legal guardians to give or refuse informed consent for incapacitated patients, considering their wishes, to protect their health and dignity. If the guardians refuse the necessary treatments in the absence of advance directives, the decision goes to the guardianship judge. The suspension of life-sustaining treatments is allowed only if the patient’s condition is irreversible and there is no chance of regaining consciousness. The patient’s will is reconstructed from their life conduct, personality, and beliefs. Guardian requests to suspend treatment must respect the patient’s right to life, regardless of health or their ability to express their will. The Italian Supreme Court of Cassation, referring to Article 2 of the European Convention on Human Rights, maintains that the right to life prevails even over the will of the guardian [63]. In Italy, the decision is shared among the physician, the trusted person, and the family members, balancing medical and moral aspects. Consequently, the importance given to the patient’s will and the degree of certainty required to suspend life-sustaining treatments are highlighted. The Italian Supreme Court of Cassation requires that the presumed will of the patient be based on clear, unequivocal, and convincing evidence. In fact, law no. 219/2017 establishes that no medical treatment can be initiated or continued without the free and informed consent of the person concerned, except for the exceptions provided by law and in compliance with the principles of the Constitution and the Charter of Fundamental Rights of the European Union [64,65]. The regulation reaffirms the importance of informed consent, allowing patients to be aware of all the information regarding their health status and the available treatments, including the benefits, side effects, and alternatives, so that they can decide whether to undergo treatment. Informed consent is essential for the effectiveness of advance healthcare directives, as defined by Article 4 of the law. Advance healthcare directives are fundamental in the therapeutic relationship, because they allow patients to indicate to physicians the course of action in the event of conditions that may result in the inability to self-determine. Through these advance healthcare directives, patients express their will regarding medical treatments, including consent to or refusal of diagnostic tests, therapeutic choices, and individual medical treatments. Advance healthcare directives can only be issued by an adult capable of understanding and willingness, after having acquired adequate medical information on the consequences of their choices. Consent must be complete, effective, and informed and the physician must demonstrate having fulfilled this obligation in case of a dispute by the patient [66]. A thorough understanding of one’s clinical case, obtained through accurate information provided by the medical team, is necessary to enable the patient to make informed decisions according to their beliefs and will. Although advance healthcare directives and informed consent are related, the physician’s duty to inform is not limited to the effectiveness of advance healthcare directives. Informed consent is a prerequisite for the lawfulness of medical–surgical treatment and protects the right to therapeutic self-determination, as confirmed by the Italian Supreme Court of Cassation [67]. According to the Italian regulations, the trustee is responsible for the implementation of advance directives, which can be revoked at any time. The guardianship judge intervenes in the case of conflict between the physician and the trustee. Advance directives are binding, but the physician may deviate from them with the trustee’s agreement if they are incongruous or do not correspond to the current clinical condition or if new therapies emerge that were not foreseeable at the time of signing and offer the possibility of improvement [68]. This exception balances healthcare autonomy and the patient’s right to self-determination. Advance directives must be drawn up in a public deed or an authenticated private writing and delivered to the civil status office. The notary ensures the patient’s freedom and medical information. Advance directives can be recorded on video if the patient is unable to draft them in the traditional manner.

### 3.12. Luxembourg

In Luxembourg, end-of-life legislation is particularly advanced and comprehensive. Since 2009, with the adoption of the “Loi du 16 mars 2009 sur l’euthanasie et l’assistance au suicide” [69], active euthanasia has been legalized for adult patients. The legislation allows adult patients suffering from severe and incurable conditions, which cause constant and unbearable physical or psychological suffering, without the prospect of improvement, to request euthanasia. Requests must meet stringent criteria to ensure that the decision is well considered and voluntary [70]. Assisted suicide is similarly regulated by the same 2009 law. As with active euthanasia, patients must meet specific criteria to access this practice. The physician must ensure that the patient understands all the implications and that the choice is free and informed, thoroughly documenting consultations and decisions in the patient’s medical dossier [71]. Advance directives are legally recognized in Luxembourg [72]. These allow individuals to express their preferences regarding medical treatments and end-of-life decisions in advance, in case they are no longer able to communicate them in the future. The directives are valid for 5 years and are renewable.

## 4. Discussion

The problems of the end of life, rather than undergoing a legislatively oriented evolution, have been the subject of a significant jurisprudential evolution at a European level [73]. In a context dominated by the absence of a common consensus regarding the recognition of a right to die, the European Court of Human Rights (ECtHR), respecting the limits of competence left to the member states, has attempted to resolve the complex issues concerning the end of life according to the principles of fundamental human rights. The review of European legislation carried out in this study has highlighted that there is no explicit regulatory provision of a conventional right to die, which, therefore, does not receive express protection in the European conventional sphere. Conversely, in the modern era, it is the interpretative dynamism of the ECtHR that has made it possible to progressively establish the right to die a death worthy of a human being, starting from an extended conception of individual freedom [74]. The examination of European regulations on end-of-life issues has highlighted the disparities and points of convergence in individual legislations. For this study, only selected European countries were examined to offer a representative sample. This selection was made to ensure that the analysis could provide a balanced view of the diverse approaches taken by various legal systems within Europe. By focusing on a subset of countries, this study aims to capture the essence of the different legal, ethical, and cultural considerations that influence end-of-life legislation across the continent. This approach allows for a more manageable and in-depth exploration of the topic, highlighting the key trends and differences that might be lost in a broader analysis of all European countries. The selected countries represent a mix of those with progressive legislation on euthanasia and assisted suicide and those with more restrictive or prohibitive approaches. From a European perspective, it would be desirable to develop supranational regulations that, while respecting the legal systems of individual countries, as well as the European Convention on Human Rights and Fundamental Freedoms, could standardize the methods of managing end-of-life care. This is also to prevent patients from moving between nations in search of legislation that is most suited to their end-of-life wishes and that fits their clinical conditions. What consistently emerges in the analysis of European legislations is the importance of self-determination in the medical field and the management of health and life by patients, even though the issue presents specific profiles in each individual situation. A common European regulation cannot disregard the reasons adopted by the ECtHR in individual cases brought to its attention, which should serve as a reference point for the development of national laws compatible with the European Convention on Human Rights and Fundamental Freedoms. Most of the decisions by the ECtHR concern the right to respect for private life and the prohibition of discrimination (Articles 8 and 14) [75]. A distinction must be made between cases where the right to end one’s life has been invoked through the cessation of life-sustaining treatments or the ingestion of lethal substances (possibly assisted by others or facilitated by actions requested from public authorities). These differ from those in a hospital setting where treatments are discontinued for patients who are artificially kept alive and are in a state of incapacity to understand and decide. These are different situations because, in the first case, there is a current decision to die by the person themselves, while, in the second case, only the cessation of life-sustaining treatments is requested for patients who are not able to give consent at that moment [76]. It emerges from the examination of European legislations that in the case of an adult patient capable of understanding and deciding, the theme of personal autonomy and the right to respect for it prevails. The right to personal self-determination is the common element consistently recognized by national legislations, as well as the basis for the interpretation of Article 8 of the European Convention, which provides for the right to respect for private and family life [77]. Therefore, a person can refuse to consent to treatments that could prolong their life, avoiding an undignified end of life. Indeed, “The dignity and freedom of man are the very essence of the Convention. Without in any way denying the principle of the sanctity of life protected by the Convention, the Court considers that the notion of quality of life takes its meaning within the framework of Article 8 of the Convention…”; provided that the person is able to freely decide on the manner and timing of the end of their own life, there is a right based on Article 8 of the Convention [78]. Since it is a right derived from Article 8 of the Convention, it not only cannot be denied by domestic law but must indeed be guaranteed through effective recourse to the judiciary [79]: it is therefore considered that the states are obliged to intervene to enable individuals to realize the object of their autonomous decisions (positive obligations), albeit within the limits imposed by the need to ensure that decisions to end one’s life are not hasty, flawed, or unfree. The development of common legislation appears desirable. Indeed, the characteristics of some national legal frameworks highlight the conflict with what is derived from Article 8 of the Convention, namely, the respect for autonomy and thus the possibility that the respect for decisions autonomously made by patients is upheld.

From a clinical point of view, which is almost unanimous and, in our opinion, justifiable, there is a prohibition on active euthanasia, understood as actions by the physician (or another person) aimed at directly causing the patient’s death. However, those procedures that are necessary to ensure the performance of the patient’s vital functions, to the extent that their omission or interruption would predictably result in the patient’s death within a short period, must certainly be considered life-sustaining treatments. These can be legitimately refused by the patient, who already has, in this way, the right to expose themselves to an imminent risk of death because of this refusal [80].

In this case, it is unreasonable for the criminally sanctioned prohibition of assisted suicide to continue to operate. Moreover, individual national legislations already provide that interventions aimed at shortening life and suffering may only take place in the case of an incurable disease and the persistence of the conditions of full capacity of the patient, incompatible with any possible psychiatric pathology, and also the presence of intolerable suffering, whether physical or “existential” [81].

Such suffering, moreover, may prove refractory to any palliative therapy, as continuous deep sedation cannot be considered a practicable alternative for patients who are not yet in terminal condition or who, in any case, refuse such treatment. The cases that have been dealt with over the years by the ECtHR highlight the lack of consensus among the various legal systems. This is an undeniable fact: in matters of the end of life, there is a significant difference of views in Europe. There are countries, such as Italy, where the regulation on living wills has only recently been achieved [60]; others have practiced assisted suicide for several years (Switzerland, the Netherlands, and Belgium); and some even allow euthanasia for terminally ill minors (Belgium and Netherlands), albeit under very specific conditions. Among these countries, Switzerland is notable as the only nation that explicitly allows non-residents to access assisted dying services, leading to what is sometimes called “suicide tourism”. While Belgium and the Netherlands technically do not prohibit foreigners from accessing their services, in practice they generally require patients to establish a relationship with a local physician over time, making it more difficult for non-residents to access these services.

A common legislation, at least regarding the fundamental aspects of the problem, particularly the definitional ones, would be desirable. While respecting the line indicated by the ECtHR, the definition of the margin of discretion available to states cannot be made without an analysis of the centrality of the end-of-life issue concerning the considered right. Common legislation should recognize the centrality of the right to private life and the individual perception of health, well-being, and quality of life [82].

Otherwise, precedence would be given to the perceptions and ethical sentiments of the majority within the legal systems, in a matter where, instead, moral pluralism should find appropriate guarantees, considering the concept of a democratic society as adopted by the Court itself.

One of the key strengths of this study is its unique nature in providing a comprehensive comparison of the various characteristics and critical aspects of end-of-life legislation across Europe. This comparative approach offers valuable insights into the similarities and differences in legislative frameworks, providing a thorough understanding of the legal landscape in this sensitive and important area.

A notable limitation of this study is the rapidly evolving nature of jurisprudence, which makes it challenging to maintain an always up-to-date perspective. Legislative changes and judicial interpretations can occur frequently, potentially affecting the relevance and accuracy of this study’s findings over time. This dynamic legal environment necessitates continuous monitoring and updates to ensure this study remains current and reflective of the most recent legal developments.

## 5. Conclusions

The research on end-of-life legislation in Europe reveals diverse cultural, ethical, and legal perspectives, impacting the accessibility and legality of medical assistance in voluntary death. Although comprehensive legislation for those not on life-sustaining treatments is lacking, progress has been made in recognizing rights in irreversible health situations, as seen in France. The development of medical technologies has brought ethical, moral, and legal needs to the forefront of the end-of-life debate.

These advances have led to the prolongation of life even in precarious conditions, raising numerous debates and profoundly changing our sensitivity towards suffering, death, and the dignity of living. Technological progress has saved lives and enhanced quality of life, yet it has also prolonged suffering for some patients without hope of recovery. Consequently, there is increasing support for laws permitting medical assistance in voluntary death to uphold the right to a dignified end. End-of-life issues intertwine with autonomy and self-determination; many assert that individuals should have the right to make their own life and death decisions without external interference, as this right is integral to human dignity.

Significant progress has been made in Europe in recognizing the right to die with dignity, but universal access to medical assistance in voluntary death is not guaranteed. This leads to ongoing debates on the ethical, moral, and legal needs of society. The European Court of Human Rights (ECtHR) supports the need for common and uniform legislation to protect every individual’s ultimate right under fundamental rights. This approach ensures that every end-of-life choice is represented in the law, reflecting a choice between different ways of dying rather than a choice between life and death. The ECtHR recognizes the diversity of legal cultures on euthanasia, allowing the practices that align with fundamental human principles and oppose therapeutic obstinacy conflicting with human dignity.

The differences in end-of-life laws have led to patient migrations seeking favorable regulations, emphasizing the need for understanding the legislative frameworks’ influence on decisions and care access. Future research should focus on evolving legislation, tracking changes, and conducting comparative studies on the legal frameworks’ effectiveness in protecting patient rights and autonomy. Harmonized European legislation is needed to ensure equitable access, uphold human dignity, and emphasize autonomy and self-determination as fundamental rights in end-of-life decisions.

## Figures and Tables

**Table 1:** European legal systems analyzed.

Country	Active Euthanasia	Euthanasia on Minors	Advance Directives	Assisted Suicide
Austria	Forbidden	Forbidden	Allowed	Allowed
Belgium	Allowed	Allowed	Allowed	Allowed
Bulgaria	Forbidden	Forbidden	Forbidden	Forbidden
Cyprus	Forbidden	Forbidden	Forbidden	Forbidden
Croatia	Forbidden	Forbidden	Forbidden	Forbidden
Denmark	Forbidden	Forbidden	Allowed	Forbidden
Estonia	Forbidden	Forbidden	Forbidden	Forbidden
France	Forbidden	Forbidden	Allowed	Forbidden
Germany	Forbidden	Forbidden	Allowed	Allowed
Greece	Forbidden	Forbidden	Forbidden	Forbidden
Italy	Forbidden	Forbidden	Allowed	Forbidden
Luxembourg	Allowed	Forbidden	Allowed	Allowed
Netherlands	Allowed	Allowed	Allowed	Allowed
Portugal	Allowed	Forbidden	Allowed	Allowed
Spain	Allowed	Forbidden	Allowed	Allowed
Switzerland	Forbidden	Forbidden	Allowed	Allowed
United Kingdom	Forbidden	Forbidden	Allowed	Forbidden

## Data Availability

Data are contained within the article.
